# Absence of *TERT* promoter mutations in colorectal precursor lesions and cancer

**DOI:** 10.1590/1678-4685-GMB-2017-0133

**Published:** 2018-02-19

**Authors:** Adriana Cruvinel-Carloni, Letícia Yamane, Cristovam Scapulatempo-Neto, Denise Guimarães, Rui Manuel Reis

**Affiliations:** 1 Molecular Oncology Research Center, Hospital de Câncer de Barretos, Barretos, SP, Brazil; 2 Department of Pathology, Hospital de Câncer de Barretos, Barretos, São Paulo, Brazil; 3 Department of Endoscopy, Hospital de Câncer de Barretos, Barretos, São Paulo, Brazil; 4 Life and Health Sciences Research Institute (ICVS), School of Health Sciences, University of Minho, Braga, Portugal; 5 ICVS/3B’s - PT Government Associate Laboratory, Braga/Guimarães, Portugal

**Keywords:** colorectal carcinoma, *TERT* promoter mutations, precursor lesions

## Abstract

Hotspot mutations (c.-124bp G > A and c.-146bp G > A) in the promoter region of the *TERT* gene have been recently described in several types of solid tumors, including glioma, bladder, thyroid, liver and skin neoplasms. However, knowledge with respect to colorectal precursor lesions and cancer is scarce. In the present study we aimed to determine the frequency of hotspot *TERT* promoter mutations in 145 Brazilian patients, including 103 subjects with precursor lesions and 42 with colorectal carcinomas, and we associated the presence of such mutations with the patients clinical-pathological features. The mutation analysis was conclusive in 123 cases, and none of the precursor and colorectal carcinoma cases showed *TERT* promoter mutations. We conclude that *TERT* mutations are not a driving factor in colorectal carcinogenesis.

## Introduction

Colorectal cancer (CRC) is the third most frequent type of cancer worldwide (Ferlay *et al.,* 2015). This scenario shows the importance to improve strategies for CRC prevention and early detection to decrease its incidence and mortality (Goss *et al.,* 2013). CRC arises from a stepwise evolution of normal mucosa to precursor lesions and ultimately to a malignant tumor. The adenoma is the most commonly reported precursor lesion of CRC (Fearon and Vogelstein, 1990; Lieberman *et al.,* 2000). However, alternative precursor lesions include the serrated polyp, which was recently described as a precursor lesion of CRC. Serrated polyps are known to be a heterogeneous group of colorectal lesions that include hyperplasic polyps (HPs), sessile serrated adenoma (SSA), traditional serrated adenoma (TSA) and mixed polyps. Clinically, HPs are the most common precursor serrated lesions of CRC (Yamane *et al.,* 2014). Serrated adenocarcinomas accounts for about 10% of all CRCs (Makinen, 2007).

The classic genetic model for colorectal tumorigenesis is driven by the progressive accumulation of a series of critical mutations in cancer-related genes, such as APC and *KRAS* (Fearon and Vogelstein, 1990). Since the molecular alterations among serrated pathways are less understood, the *BRAF* gene has now emerged as a prevalent marker in this pathway (Yamane *et al.,* 2014). With the presence of these genetic alterations, molecular biomarkers have been widely proposed as a means of CRC screening and prevention (Imperiale and Ransohoff, 2010).

Recently, hotspot somatic mutations in the *TERT* promoter region (c.-124bp G > A and c.-146bp G > A) have been described in several tumors, particularly skin, brain, thyroid and bladder cancers (Horn *et al.,* 2013; Huang *et al.,* 2013; Killela *et al.,* 2013; Vinagre *et al.,* 2013; Heidenreich *et al.,* 2014). The *TERT* gene encodes a telomerase reverse transcriptase, an essential protein for preserving telomere genomic integrity. These mutations result in the creation of new binding motif sites (GGAA) for ETS transcription factors, leading to an increase in *TERT* activity and subsequent telomere preservation (Horn *et al.,* 2013; Huang *et al.,* 2013). Additionally, these hotspot mutations have been associated with advanced tumor stages and poor prognosis for patients (Killela *et al.,* 2013; Vinagre *et al.,* 2013; Heidenreich *et al.,* 2014). Currently, only one study evaluated *TERT* mutation frequency in CRC, and no mutations were found in colorectal adenocarcinomas (Killela *et al.,* 2013).

Herein, we investigated the frequency of *TERT* mutations in a series of Brazilian patients with colorectal precursor and cancer lesions. We analyzed 145 Brazilian patients from the Barretos Cancer Hospital. The clinico-pathological and molecular features of the patients were previously reported (Table 1) (Yamane *et al.,* 2014). All included patients were over 50 years old, with a mean age of 66 years (ranging from 51 – 89), with similar frequency for both genders. Patients with known family history, hereditary CRC, or bowel inflammatory disease were excluded. All cases were reviewed by an expert pathologist and categorized according to the WHO classification. Tumor DNA was isolated from formalin-fixed paraffin-embedded (FFPE) tumor tissue, as previously reported (Yamane *et al.,* 2014). *TERT* promoter mutations were identified by PCR followed by direct sequencing as described elsewhere (Vinagre *et al.,* 2013; Batista *et al.,* 2016).

**Table 1:** Clinicopathological and molecular features of all patients.

Variables		N	%
Age	66.0 y mean (range 51- 89)	145	-
Gender	Female	71	49.0
	Male	74	51.0
Histology	Adenocarcinoma	42	29.0
	Adenoma polyps	50	34.5
	Serrated polyps	15	10.3
	Hyperplastic polyps	38	26.2
Precursor Lesion Location	Right colon	39	37.9
	Left colon	64	62.1
Carcinoma Location	Right colon	24	57.1
	Left colon	18	42.9
Precursor Lesion Morphology	Polypoid	83	81.4
	Non polypoid	19	18.6
Precursor Lesion Size (mm)	< 10	91	90.1
	≥ 10	10	9.9
Precursor Lesion MSI Status	MSS	96	96.0
	MSI-L	4	4.0
	MSI-H	0	0.0
Carcinoma MSI Status	MSS	35	83.3
	MSI-L	2	4.8
	MSI-H	5	11.9
Precursor Lesion *KRAS* Status	MUT	14	13.6
	WT	89	86.4
Precursor Lesion *BRAF* Status	MUT	9	8.7
	WT	94	91.3
Carcinoma *KRAS* Status	MUT	20	47.6
	WT	22	52.4
Carcinoma *BRAF* Status	MUT	2	4.8
	WT	40	95.2

MUT, mutated; WT, wild type; MSI-L, microsatellite instability low; MSI-H, microsatellite instability high; MSS, microsatellite stability.

**Figura 1:**
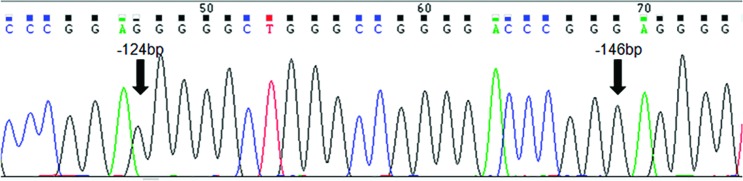
Electropherogram of *TERT* showing the wild-type sequence for both hotspot mutation regions. The arrows indicate the hotspot mutation regions (-124bp and -146bp).

Of the 145 samples analyzed, 22 were inconclusive due to poor quality/quantity of DNA. The evaluation of hotspot *TERT* promoter mutations showed that all precursor and cancer lesions (123 samples), which included 45 adenoma polyps, 15 serrated polyps, 22 hyperplastic polyps and 41 adenocarcinomas, were wild-type (Figure 1). Our results are in agreement with a previous report that showed the absence of *TERT* promoter mutation in colorectal adenocarcinomas (Killela *et al.,* 2013). We also showed for the first time, that these mutations are absent in precursor lesions as well. Furthermore, it is the first study to analyze the *TERT* mutation status in Brazilian colorectal disease patients.

Telomere length is a major tumor hallmark (Heidenreich *et al.,* 2014). Besides hotspot *TERT* promoter mutations, other pathways are involved with an increase in telomere length (Heidenreich *et al.,* 2014). One such mechanism is the alternative lengthening of telomeres (ATL) (Cesare and Reddel, 2010; Killela *et al.,* 2013). However, a previous study reported the absence this pathway in CRC (Heaphy *et al.,* 2011). Therefore, the mechanisms of telomere length variation in colorectal tumors are still unknown.

Concluding, we analyzed for the first time the presence of *TERT* promoter mutations in precursor and carcinoma colorectal lesions in Brazilian patients. The results showed the lack of *TERT* promoter mutations, suggesting that these alterations are not involved in CRC carcinogenesis.
